# Strategies to Investigate Membrane Damage, Nucleoid Condensation, and RNase Activity of Bacterial Toxin–Antitoxin Systems

**DOI:** 10.3390/mps4040071

**Published:** 2021-10-08

**Authors:** Stefano Maggi, Alberto Ferrari, Korotoum Yabre, Aleksandra Anna Bonini, Claudio Rivetti, Claudia Folli

**Affiliations:** 1 Department of Chemistry, Life Sciences and Environmental Sustainability, University of Parma, 43124 Parma, Italy; smaggi@caltech.edu (S.M.); aleksandraanna.bonini@unipr.it (A.A.B.); 2 Department of Food and Drug, University of Parma, 43124 Parma, Italy; axf1301@miami.edu (A.F.); korotoum.yabre@studenti.unipr.it (K.Y.)

**Keywords:** toxin–antitoxin systems, ribonuclease toxins, membrane-associated toxins, inhibition growth assays, single-cell fluorescence microscopy, atomic force microscopy

## Abstract

A large number of bacterial toxin–antitoxin (TA) systems have been identified so far and different experimental approaches have been explored to investigate their activity and regulation both in vivo and in vitro. Nonetheless, a common feature of these methods is represented by the difficulty in cell transformation, culturing, and stability of the transformants, due to the expression of highly toxic proteins. Recently, in dealing with the type I Lpt/RNAII and the type II YafQ/DinJ TA systems, we encountered several of these problems that urged us to optimize methodological strategies to study the phenotype of recombinant *Escherichia coli* host cells. In particular, we have found conditions to tightly repress toxin expression by combining the pET expression system with the *E. coli* C41(DE3) pLysS strain. To monitor the RNase activity of the YafQ toxin, we developed a fluorescence approach based on Thioflavin-T which fluoresces brightly when complexed with bacterial RNA. Fluorescence microscopy was also applied to reveal loss of membrane integrity associated with the activity of the type I toxin Lpt, by using DAPI and ethidium bromide to selectively stain cells with impaired membrane permeability. We further found that atomic force microscopy can readily be employed to characterize toxin-induced membrane damages.

## 1. Introduction

Numerous efforts have been addressed to understand the mechanism of action of different toxin–antitoxin (TA) systems identified in various bacterial strains. TA systems consist of two genetic elements, the toxin capable of interfering with essential cellular processes and the counterpart antitoxin which inhibits toxin activity. TA systems are widespread in bacteria, both in chromosomes where they can be present in multiple copies, and in mobile elements [1]. While it is well documented that stress conditions can trigger the TA loci transcription [2,3], the role of chromosomal TA systems in host bacterial physiology is still debated [4,5]. In a recent classification, TA systems are grouped into 8 classes (type I-VIII) based on the molecular nature of the antitoxin and its action mechanism [6,7].

In type I TA systems, the antitoxin is a sRNA that binds the mRNA coding for the toxin peptide resulting in translation inhibition. Type I toxins are peptides or small proteins (less than 60 aa) containing a potential trans-membrane domain and they have been associated with membrane depolarization, ATP leakage, and with nucleoid condensation, with the exception of SymE and RalR which act as RNase and DNase, respectively [8,9,10].

In type II TA systems, an unstable antitoxin protein inhibits the activity of the stable toxin protein by complex formation. Numerous studies have been reported on type II toxins acting as ribonucleases, such as MazF, RelE, and YafQ. Other activities have been associated with these toxic proteins, such as inhibition of replication or inhibition of peptidoglycan synthesis [11].

Type III TA systems are characterized by a toxin protein and an antitoxin sRNA capable of neutralizing the toxin by direct binding, while type IV TA systems are regulated by the toxin–antitoxin binding competition versus the same molecular target [7]. The only member of the type V group is GhoST, in which the antitoxin (GhoS) is a ribonuclease that specifically cleaves GhoT mRNA [12]. Type VI is associated with socAB TA system, characterized by an unstable toxin protein constitutively degraded by the protease ClpXP which requires the antitoxin as a proteolytic adaptor [13]. In type VII TA systems, the antitoxin is an enzyme with the ability to inactivate the toxin by oxidizing cysteine residues [14]. Very recently, a new type VIII TA system has been proposed, in which an RNA toxin is inhibited by an RNA antitoxin through antisense binding [15].

In the last fifteen years, the study of toxin–antitoxin (TA) systems has attracted the interest of numerous research groups working on molecular mechanisms of prokaryotes, and different in vitro and in vivo experimental approaches have been used to investigate the activity of toxins, antitoxins, and their complexes. However, the majority of the available experimental data regards primarily type I and type II TA systems, making them the preferred candidates for in silico identification of new TA systems [16].

Within this context, we recently reported the identification in several *Lactobacillus* strains of the type I TA system Lpt/RNAII and different type II DinJ-YafQ systems [17,18,19], for which we developed experimental strategies aimed to characterize their activity both in vivo and in vitro. In this article we collectively describe these strategies by reporting simple protocols for culture growth, protein expression, and toxicity assessment in the bacterial host *Escherichia coli*.

## 2. Materials and Methods

### 2.1. Materials

Media, buffer components, standard reagents, and the fluorescence probes Thioflavin-T (ThT) and 4′,6-diamidino-2-phenylindole (DAPI) were obtained from Sigma Aldrich (Darmstadt, Germany); ethidium bromide (EB) was purchased from Promega (Madison, WI, USA) and TALON metal affinity resin functionalized with Co^2+^ from Takara (Kusatsu, Shiga, Japan). Poly-L ornithine was purchased from Sigma Aldrich. Phosphate-buffered saline (PBS) was purchased from VWR and contained 137 mM sodium chloride, 2.7 mM potassium chloride, and 10 mM phosphate buffer pH 7.3–7.5.

### 2.2. Bacterial Strains and Expression Vectors

pSRKKm plasmid was a kind gift of Stephen K. Farrad; pET11b and pET28b expression vectors and *E. coli* BL21(DE3) cells were purchased from Merck (Kenilworth, NJ, USA). *E. coli* C41(DE3), and C41(DE3) pLysS cells were obtained from Lucigen (Madison, WI, USA). *Lacticaseibacillus rhamnosus* strain 1019 and *Lacticaseibacillus paracasei* strain 4366, belonging to the University of Parma Culture Collection (UPCC), were isolated from dairy matrices and grown in Man Rogosa Sharpe (MRS) medium Oxoid (Waltham, MA, USA) under anaerobiosis (AnaeroGen, Oxoid) at 37 °C.

### 2.3. Cloning, Transformation of E. coli Cells, and Culture Conditions

Lpt coding sequence (GenBank ID KY321384) was PCR-amplified from total DNA of *Lacticaseibacillus rhamnosus* strain 1019 and cloned into pET11b vector. DinJ and YafQ coding sequences (GenBank ID MK544944) were PCR-amplified from total DNA of *Lacticaseibacillus paracasei* strain 4366 and cloned in pET11b and pET28b vectors, respectively.

Total DNA extraction was performed from 1 mL of overnight culture prepared as described in 2.2 using DNeasy Blood and Tissue kit (Qiagen, Hilden, Germany), following manufacturer’s instructions for lysis of Gram+ bacterial cells.

The amplification reaction was carried out under standard conditions by employing GoTaq DNA polymerase (Promega), a NdeI-tailed upstream primer and a BamHI-tailed downstream primer [17,20]. The amplification products were first cloned into the pGem-Teasy vector (pGEM^®^-T Easy Vector Systems, Promega) and then subcloned into the NdeI/BamHI restriction sites of the pET vector by using T4 DNA Ligase (New England BioLabs, Ipswich, MA, USA) according to the manufacturers’ protocol.

*E. coli* cells were transformed by electroporation using a Micropulser Electroporation Apparatus (BIORAD, Hercules, CA, USA) and selected on LB solid medium containing 100 μg/mL ampicillin or 50 μg/mL kanamycin for pET11b and pET28b, respectively. In addition, 34 μg/mL chloramphenicol was used when transforming *E. coli* C41(DE3) pLysS cells. Cells were grown in liquid LB medium at 37 °C under gentle shaking. Cell density was monitored by absorbance measurements at 600 nm.

### 2.4. Instruments

Absorbance measurements were carried out by using a Varian Cary 1E UV-visible spectrophotometer. Fluorescence microscopy analysis was performed with a Nikon Eclipse E600 equipped with a 100× immersion objective, a tungsten halogen light source, and a Niko DS-Fi2 digital camera. For DAPI/EB staining the UV-2A filter (excitation filter 355/50) was used, while for ThT staining the B2A filter (excitation filter 470/40) was used. All fluorescence images were captured in the RGB color space, with a size of 2560 × 1920 pixels and a spatial resolution of 52 nm/pixel. Atomic force microscopy imaging was conducted, with a Park XE-100 microscope (Park Systems, Suwon, Korea) operating in intermittent mode with a scan rate of 0.5 Hz. Commercial diving board silicon cantilevers (MikroMasch, Sofia, Bulgaria) were used. Milli-Q water (Millipore, Burlington, MA, USA) was used to prepare all samples for microscopy.

### 2.5. Software

Images were processed with ad-hoc scripts written Matlab (The MathWorks, Inc., Natick, MA, USA) with Image processing toolbox. Images were displayed either with the Matlab graphical interface or with Gwyddion software (http://gwyddion.net, accessed on 12 July 2019).

## 3. Results and Discussion

### 3.1. Strains and Vectors Suitable for Toxin Activity Studies

The in vivo activity of bacterial toxins is generally evaluated by inhibition growth assays conducted in liquid or on solid media, and *E. coli* host cells are usually chosen for their ease and rapid growth and for the plethora of expression systems commercially available [17,21]. However, because of the high cell toxicity of toxin products, the choice of the expression vector and of the host bacterial strain is crucial for an in vivo analysis. For instance, to evaluate the effect of a toxin on cell viability it is necessary to employ a highly repressed and tightly controlled expression system to allow growth of the bacterial host prior to toxin induction. mRNA leakage of the expression system due to basal transcription is detrimental to cell growth and results often in the selection of mutated or undesirable clones [17]. The pET system is often used for the expression of recombinant proteins in *E. coli*. Target ORFs are cloned in a multiple cloning site of a pET plasmid (copy number 15–20) under the control of the strong T7 promoter. Expression of the recombinant protein is achieved by means of a T7 RNA polymerase encoded by a gene integrated in the bacterial chromosome under the control of the PlacUV5 promoter. Both the T7 and PlacUV5 promoters harbor a lac operator sequence for *lac*I mediated repression and isopropyl-1-thio-β-D-galactopyranoside (IPTG) induction [22].

Although this system guarantees sufficient repression for most recombinant proteins, in the case of bacterial toxins expressed in the absence of the corresponding antitoxin, it results in a strong growth inhibition before induction, a condition that favors the appearance of mutated phenotypes with point mutations in key residues of the toxin [17]. In these cases, a tightly repressed expression of the toxic protein can only be achieved by using *E. coli* strains expressing the T7 lysozyme (pLyS strains) that down regulates transcription by binding to the T7 RNA polymerase [23], and harboring a weakened placUV5 promoter variant (C41) or a *lac*I variant with low IPTG affinity (C43) (Walker strains) [24].

In experiments that aimed to validate in vivo the activity of the type I toxin Lpt identified in *Lactobacillus rhamnosus*, we first attempted to study the toxicity of the Lpt toxin in *E. coli* by using the pSRKKm plasmid in which the expression of the cloned sequence is controlled by the IPTG inducible *lac* promoter. Although this plasmid is characterized by a very low basal expression [25], *E. coli* growth was partially inhibited also under non-induction conditions [18] and the clones isolated in few cases did not show the expected toxic phenotype upon IPTG induction. Conversely, cloning the Lpt coding sequence into a pET11 vector and subsequent transformation of *E. coli* C41 (DE3) pLysS cells allowed us to eliminate the toxic effect of the basal expression and to obtain the expected phenotype upon IPTG induction [20]. Remarkably, the toxic phenotype observed in *E. coli* C41 cells upon induction disappeared after few days from transformation with the recombinant pET plasmid, probably due to the selection of clones with mutations affecting the expression or activity of the toxin [26]. This phenomenon was not investigated further, but we managed to overcome the phenotype loss by using freshly transformed cells in each experiment. Thus, the use of colonies kept on solid media or even in frozen glycerol stocks is not recommended.

Likewise, the RNase activity of the type II toxin YafQ isolated from *Lactobacillus rhamnosus* could only be studied in *E. coli* C41 (DE3) pLysS host cells, transformed with a pET vector carrying the YafQ coding sequence. Under these conditions, it has been possible to obtain standard growth curves in the absence of IPTG and inhibited growth curves upon induction [17]. However, despite the strong inhibition of the basal expression, different types of YafQ mutants were observed with a high frequency. In particular, *wild type* YafQ toxin from *L. rhamnosus* 2360 was never isolated due to the recurrence of mutations such as frameshift deletions, insertions or substitutions mostly involving catalytic residues [17].

To carry out in vivo toxicity assays, *E. coli* cells transformed with the recombinant plasmid were grown in liquid media until an OD_600 nm_ of 0.2–0.6 was reached. Thereafter, IPTG was added to induce toxin expression and the growth curve was obtained by measuring the OD_600 nm_ over time or by counting the colony forming units per milliliter (CFU/mL) on solid medium. In the first case, a stable OD_600 nm_ value over time indicates the arrest of bacterial growth, while the calculation of CFU/mL value allows to quantify the fraction of viable cells. With this method it was possible to distinguish between growth arrest and cell death by comparing the OD_600 nm_ value and the CFU/mL for the same bacterial culture during the expression of the toxin.

Thus, the use of a pET system, combined with the *E. coli* host strain C41 (DE3) pLysS is, in our opinion, a valuable and cost-effective strategy to deeply control the expression of protein toxins.

### 3.2. Production of Purified Toxins

**Figure 1:**
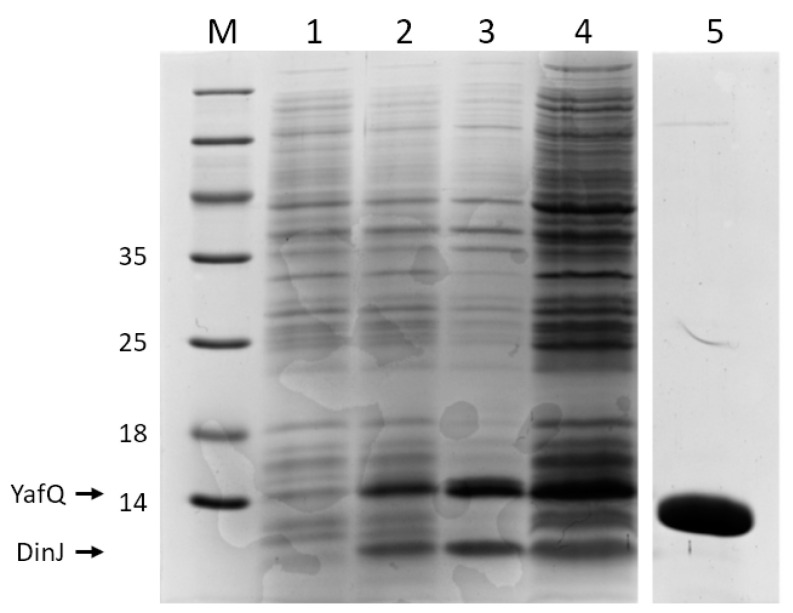
Coomassie Blue stained SDS–PAGE analysis of YafQ(His6) protein production. Lanes M, molecular mass standards (kDa); lane 1, not induced total *E. coli* BL21(DE3) cell extract; lane 2, IPTG induced total *E. coli* BL21(DE3) cell extract; lane 3, insoluble fraction after cell lysis; lane 4, soluble fraction after cell lysis; lane 5, eluted YafQ(His6) under denaturing conditions (8 M urea).

To investigate the toxin activity in vitro, the purified protein toxin is required. In the case of type I TA systems, in which the toxin is a short peptide with less than 60 amino acids, the easiest and cost-effective way to obtain the purified toxin is to purchase the peptide from commercial synthesis services, which also offers modifications with different adducts such as fluorescent probes or lipids. Conversely, the recombinant expression of type II toxins is challenging, primarily because of the high protein toxicity, and secondly because when co-expressed with the antitoxin to mitigate toxicity, the dissociation of the toxin–antitoxin complex is difficult. In studies aimed to characterize YafQ toxins identified in lactic acid bacteria, we cloned the YafQ coding sequence in the expression vectors pET11b or pET28b that were used to transform different *E. coli* strains (BL21(DE3), C41 (DE3), and C41 (DE3) pLysS). Upon induction, we could not detect the production of the wild-type protein toxins by SDS-PAGE in any of the recombinant clones that were obtained. A high expression yield of YafQ could be obtained only by the co-expression with the antitoxin DinJ in *E. coli* BL21(DE3) cells. This strategy can be achieved either by cloning the entire TA locus under the control of an inducible promoter [27,28] or by cloning the toxin and antitoxin coding sequences in different expression vectors under the control of the same or of different promoters [21,29,30]. When the toxin and the antitoxin are co-expressed in BL21(DE3) cells, high expression yields are usually observed. To obtain the purified YafQ, a tag of 6 histidines (His6) was either fused to YafQ or to the cognate antitoxin DinJ [21,27,31]. The fusion of the His6 tag to the YafQ protein increases the molecular weight difference between toxin and antitoxin, thus allowing a better separation onto the SDS-PAGE. We initially cloned antitoxin DinJ cDNA into pET11b expression vector and YafQ cDNA into NdeI restriction site of pET28b expression vector, thus adding the His6 tag to the aminoterminal of YafQ. *E. coli* BL21(DE3) cells were first transformed with pET11b-DinJ and subsequently transformed with pET28b-YafQ(His6) to obtain the double transformed strain used for YafQ isolation. Transformed cells were grown until OD_600 nm_ reached 0.6 and induced by addition of IPTG 1 mM. After 8 h of incubation at 28 °C, cells obtained from 1 L of culture were lysed by sonication in 40 mL of buffer NaH_2_PO_4_ 50 mM, NaCl 250 mM pH 7.4 and then centrifuged to separate soluble and insoluble fractions. The pellet was then solubilized in 40 mL of SDS 1% *w*/*v*. Protein samples were analyzed by SDS-PAGE under standard conditions (Figure 1). In particular, 1 mL of not induced and induced cultures was centrifuged and the pellet was resuspended in SDS 1% *w*/*v* to obtain an OD_600 nm_ of 3.3. 12 μL of these samples were loaded in lane 1 and 2 of the SDS-PAGE. 12 μL of insoluble and soluble fractions obtained after cell lysis as described above, were loaded in lane 3 and 4, respectively. The gel picture reveals the presence of both DinJ and YafQ proteins in the induced *E. coli* cells (lane 2) and in the insoluble (lane 3) and soluble (lane 4) lysed fractions. From the intensity of the bands in lane 3 and 4 we estimated a solubility of about 50% for both proteins after cell lysis. The molecular weight of the proteins is in accordance with the values of 13,605 Da for YafQ and of 9983 Da for DinJ, as provided by the Expasy Bioinformatics Portal (https://web.expasy.org/protparam/, accessed on 6 February 2021).

The complex DinJ-YafQ(His6) was purified from the soluble fraction by using a Co^2+^ functionalized column. Once bound to the column, the toxin–antitoxin complex is dissociated by overnight incubation in 100 mM Tris-HCl pH 8 containing 6 M guanidine hydrochloride. DinJ is then released in the flow-through. In a first attempt, 8 M urea was used for complex denaturation but only a partial dissociation of the DinJ-YafQ was achieved. YafQ(His6) is then eluted from the column with 100 mM Tris-HCl pH 8, 300 mM imidazole, 8 M urea. The exchange of guanidine with urea has the purpose to avoid residual guanidinium traces that could interfere with further analysis. Denatured YafQ eluted from the column is dialyzed overnight at 4 °C against the same buffer without imidazole and is then refolded by stepwise dialysis to gradually eliminate urea (Figure 1). During the renaturation step, more than 50% of YafQ precipitates as insoluble protein, thus, using these conditions only about 2 mg of YafQ(His6) were isolated per liter of cell culture. Protein concentration was determined by absorbance at 280 nm using the extinction coefficient of 22460 M^−1^ cm^−1^, as provided by the Expasy Bioinformatics Portal https://web.expasy.org/protparam/ (accessed on 6 February 2021). 14 μg of the purified YafQ was loaded in the lane 5 of the SDS-PAGE (Figure 1).

### 3.3. Fluorescence Imaging to Monitor RNase Activity

Protein toxins of type II TA systems can be classified into nine superfamilies, based on structural similarities and on the mechanism of action [32]. Four of these toxin families are RNAses that cleaves mRNA either in a ribosome dependent or independent manner. A fluorescence method for monitoring the RNA metabolism in vivo can therefore be a valuable tool for investigating the functional activity of these toxins. In 2015, Sugimoto et al. reported the use of the amyloid-binding probe ThT for monitoring RNA metabolism in vitro and in vivo [33]. ThT binds preferentially purine oligoribonucleotides, therefore it fluoresces brightly, with a peak at 491 nm, when complexed with bacterial total RNA rather than with genomic DNA. Recently, we have successfully applied ThT staining for monitoring the RNase activity of lactobacilli YafQ in vivo, upon expression of the toxin in *E. coli* C41(DE3) pLysS cells [17].

For this analysis, *E. coli* C41 (DE3) pLysS cells were grown in liquid media and YafQ expression was induced with the addition of 1 mM IPTG. After six hours of expression, an aliquot was taken from the culture; cells were washed 3 times with 1 mL of PBS and resuspended in a final volume of 50 µL of PBS. ThT was then added to a final concentration of 25 µM and incubated for 5 min at room temperature. Excess dye was removed by two washes with 1 mL of PBS and cells were resuspended in 50 µL of PBS. Finally, 25 µL of the cell suspension were deposited onto glass slides functionalized with 0.01% poly-L ornithine to promote cell adhesion. After 30 s, the coverslips were washed with Milli-Q water and dried by evaporation at room temperature. The remaining 25 µL of the cell suspension was incubated at room temperature for 20 h to allow toxin-mediated RNA degradation and further deposited onto functionalized glass slides as described. Fluorescence images were taken with a fluorescence microscope and analyzed as described below. Because of the fast ThT bleaching, cells were focused in phase contrast and fluorescence images were recorded immediately after shutter opening with an exposure time of 800 ms.

### 3.4. Image Processing

**Figure 2:**
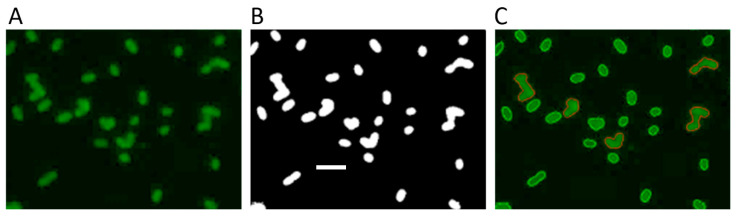
Image processing steps for the quantification of total RNA from fluorescence images of *E. coli* C41(DE3) pLys cells stained with ThT. (**A**) Green component of the raw fluorescence image. (**B**) Binarized image mask obtained using the computed global threshold. (**C**) Image segmentation showing the identified cells (green contours) and the discarded cell aggregates (red contours). Scale bar 5 μm.

The average fluorescence intensity of individual cells was determined by analyzing the images with Matlab scripts written locally (Figure 2). Because of the green ThT fluorescence, only the green component of the RGB colored images (Figure 2A) was used while the red and blue components were discarded. A global threshold was computed by using the Otsu’s method of the *graythresh* function. Images were then binarized with the *imbinarize* function using the computed global threshold to obtain the image mask (Figure 2B). Objects touching the image edge or with an area less than 100 pixels were removed by morphological image processing. This mask was then used to compute the area, perimeter, and the intensity of each object in the green component image using the *regionprops* function. To exclude cell aggregates and small debris from the analysis, objects with a computed area larger than 1000 pixels or lower than 300 pixels or with a perimeter higher than 165 pixels were discarded. The results of the image segmentation could be visually inspected by drawing the contours of the identified objects onto the original color image (Figure 2C). Because the total fluorescence intensity of each cell depends on the cell dimensions, it was normalized by the area of the cell. The normalized fluorescence intensities were used to obtain the mean fluorescence intensity of the cell sample. Comparison of the mean fluorescence intensities obtained with different samples or under different conditions provides a direct quantification of the toxin RNase activity (see Figure 4 in [17]).

### 3.5. Fluorescence Imaging to Monitor Membrane Permeabilization

The non-physiological expression of type I toxin peptides damages the bacterial cell, leading to membrane depolarization, ATP leakage, impairing of cell division, and eventually to pore formation [6]. In some cases, the membrane damage is also associated with nucleoid condensation. To investigate in vivo the detrimental effect of these membrane-active peptides, two different classes of fluorescent dyes, characterized by different membrane permeability, are usually employed. The nucleic acid staining dyes DAPI, SYTO9, fluorescein diacetate (FDA), and Acridine Orange permeate intact cell membrane and can thus be used to stain the whole bacterial population. Conversely, EB and its structural analogs Propidium Iodide (PI) and SYTOX™ Orange permeate only cells with a damaged membrane. In addition, the fluorescence dyes Bis-(1,3-Dibutylbarbituric Acid) Trimethine Oxonol (DiBAC4(3)), 3,3′-Diethyloxacarbocyanine Iodide (DiOC2(3)), and 3,3′-Dihexyloxacarbocyanine Iodide (DiOC6(3)) permeate cell membranes based on their polarization state. Commercially available kits, exploiting the combination of membrane-permeant and membrane-non-permeant nucleic acid dyes (LIVE/DEAD BacLight kit), are also available to assess the viability of bacterial cells, however they should be used with care to avoid data misinterpretation [34].

Flow cytometry studies reported in the literature show that staining *E. coli* with EB reveals one major population of cells with low fluorescence signal and a minor population of cells with a high fluorescence signal. The treatment of *E. coli* cells resuspended in PBS with the addition of EDTA, which is known to compromise membrane integrity, increases the fluorescence intensity by ten times, however, the fluorescence intensity remains about 2% of the value measured with fixed cells [35]. Furthermore, the staining efficacy of PI, commonly used as a marker to identify dead cells, and EB has been compared, using cells with dissipated membrane potential, highlighting that the membrane permeability of these two dyes depends solely on the integrity of the lipid bilayer and not on the membrane potential [36]. For these reasons, EB is a good candidate for characterizing the effect of type I toxins on membrane integrity and stability.

**Figure 3:**
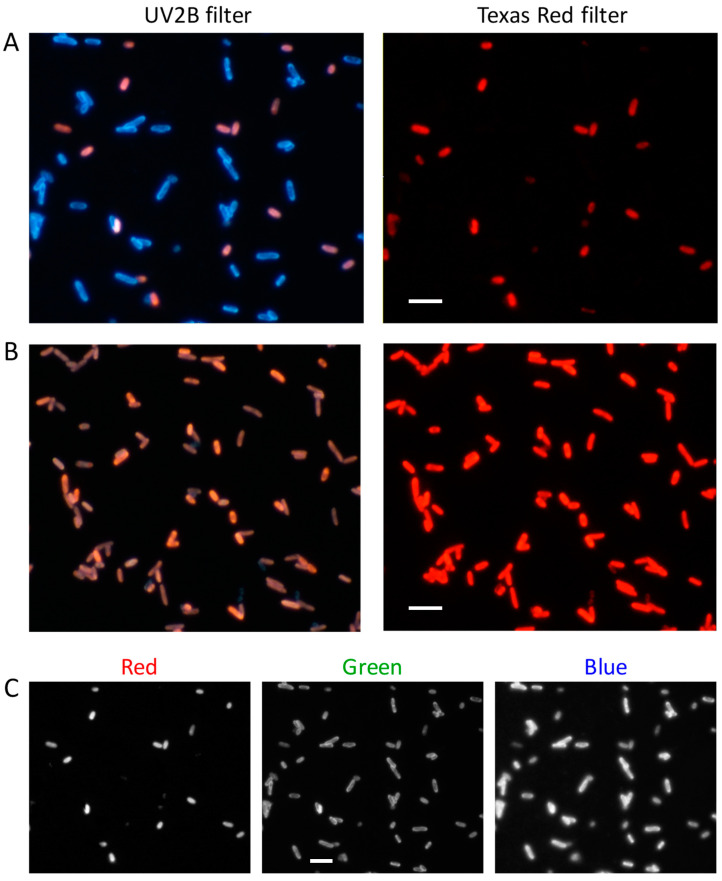
Benchmark test of the DAPI/EB staining to identify cells with impaired membrane integrity. (**A**) Fluorescence images of unfixed *E. coli* C41(DE3) pLys cells resuspended in PBS obtained with the UV2B filter (**left**) or with the Texas Red filter (**right**). (**B**) Fluorescence images of glutaraldehyde-fixed *E. coli* C41(DE3) pLys cells resuspended in PBS obtained with the UV2B filter (**left**) or with the Texas Red filter (**right**). (**C**) RGB components of the fluorescence image shown in A captured with the UV2B filter. Scale bar 5 μm.

Based on the data reported in the literature, we recently developed a fast and cost-effective staining procedure, using a combination of DAPI and EB to selectively stain *E. coli* cells with normal or altered membrane permeability. As a proof of concept, we first verified the method by using *E. coli* C41 plysS cells in stationary phase and preparing unfixed or glutaraldehyde-fixed samples as prototypes of cells with intact or impaired membrane, respectively [35]. In particular, 1 mL of cell culture in stationary phase was harvested and cells were washed three times with 1 mL of PBS and re-suspended in 50 μL of PBS. To prepare fixed samples, cells were treated with glutaraldehyde at a final concentration of 1% *v*/*v*, rotated for 1 h at room temperature, washed three times with 1 mL of PBS to remove glutaraldehyde excess and re-suspended in 50 μL of PBS. Fixed and unfixed samples were stained with DAPI/EB added at a final concentration of 10 μg/mL each, followed by 5-min incubation at room temperature, washed twice with 1 mL of PBS to remove dyes excess, then resuspended in 50 μL of PBS. An aliquot of 25 μL of unfixed and fixed cell suspensions was deposited onto a glass coverslip functionalized with poly-L ornithine and let it sit for 30 s, washed with Milli-Q water, and dried by evaporation at room temperature. Fluorescence images were taken with a fluorescence microscope equipped with a digital camera (Figure 3).

As shown in Figure 3A, most of the cells in the unfixed sample stained with DAPI/EB showed the blue fluorescence signal associated to DAPI, while a limited subpopulation was characterized by a red signal typical of EB. Conversely, in the fixed sample (Figure 3B) all the cells were stained with EB, thus confirming the different permeability of these two dyes. Under a fluorescent microscope it was possible to visualize both signals by using a UV2B filter, or to selectively visualize the EB signal by using a Texas red filter. This allowed easy discrimination between cells with damaged and undamaged membrane. Given the spectral separation of the fluorescence signals, the same result could be achieved by using the red and blue components of the RGB image obtained with the UV2B filter (Figure 3C). This latter method has the advantage to require only one image, thus avoiding image processing procedures to correct misalignments due to the filter change.

This procedure was applied to study the effects induced by the expression of the toxic Lpt peptide in *E. coli* [20]. For the analysis, a liquid medium was inoculated with a single colony of *E. coli* C41 plysS cells freshly transformed with pET11b-*lpt*. 1 mL of culture was taken during growth and, after OD_600 nm_ measurement, used to prepare glass coverslips of unfixed samples as described above. At OD_600 nm_ of 0.2, IPTG was added at a final concentration of 1 mM to induce Lpt expression. To avoid staining bias due to differences in the number of cells harvested, each aliquot of culture was normalized to an OD_600 nm_ of 0.5.

**Figure 4:**
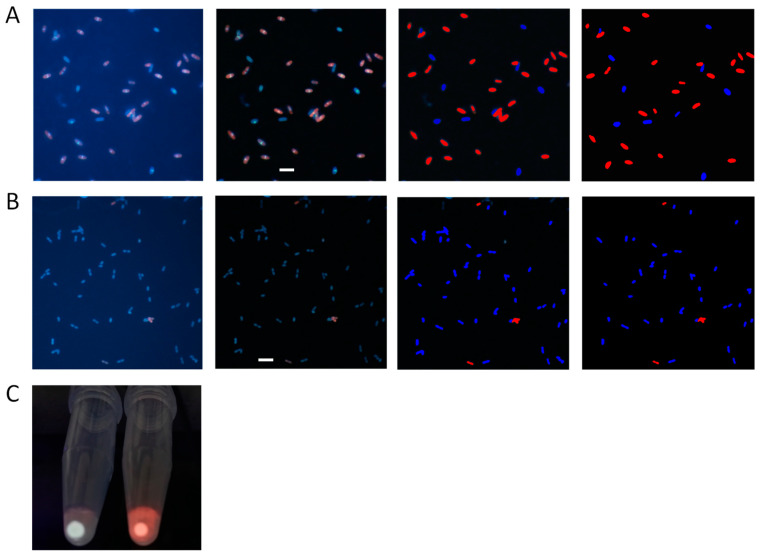
Image processing steps of induced (**A**) and not induced (**B**) *E. coli* C41(DE3) pLys transformed with pET11b-lpt vector. From left to right: raw fluorescence image captured with the UV2B filter; background subtracted image; cell color classification; and final image with segmented objects. (**C**) Tubes with pelleted *E. coli* C41 (DE3) pLys cells stained with DAPI/EB and exposed onto a UV-transilluminator (λmax 312 nm). No filters were used to generate the image. Not-induced cell culture (**left**); Cell culture expressing the membrane-active Lpt peptide (**right**). Scale bar 5 μm.

Figure 4A,B shows fluorescence microscopy images of non-induced and induced cells stained with DAPI/EB and the steps of the image processing procedure used to identify cells with damaged membrane. Beside the loss of membrane integrity, some type I toxins, including Lpt, cause nucleoid compaction that can be detected under the fluorescence microscope as discrete clear spots mostly located near the center of the bacteria (Figure 4A). This phenotype can be quantitatively assayed by determining the recurrence of this feature within the bacteria population and also by measuring morphology parameters of the nucleoid, as described below.

### 3.6. Image Processing

Hereafter, we describe the image processing procedure that can be used to quantitatively analyze the fluorescence images collected with the UV2B filter. The procedure uses the advantages of the Matlab software and the Image processing toolbox to count the number of cells with a damaged membrane (EB stained) or with an integral membrane (DAPI stained). The analysis was then extended to measurements of several morphological parameters of the cell nucleoid that could be used to reveal nucleoid compaction associated with toxin activity. Starting from the raw color image, the background was suppressed with the top–hat filtering algorithm by using an elliptical structuring element with radius 60 pixels and height 5 pixels. Instead of using directly the red and the blue component images of the filtered RGB image, the EB signal was obtained by subtracting the Blue component from the grayscale filtered image, while the DAPI signal was obtained by subtracting the Red component from the grayscale filtered image (values lower than zero are set to zero). This allowed better object recognition in the subsequent steps.

The *graythresh* function with default parameters was then used to generate the binary image mask from the EB signal (red-mask) and from the DAPI signal (blue-mask). Small objects (with less than 300 pixels) and objects touching the image edge were discarded. Because cells stained with EB can also show up in the blue component image while cells stained with DAPI never show up in the red component image, to avoid counting them twice, objects identified in both masks were deleted from the blue-mask. This was achieved by subtracting the 3 pixel dilated red-mask from the blue-mask. Thus, if a cell was revealed in both the DAPI and EB signals it was counted only for the latter signal.

The *regionprops* function was then used to measure the objects in the red-mask and in the blue- mask. To exclude cell aggregates, objects with a computed area larger than 2000 pixels or lower than 400 pixels or with a perimeter higher than 250 pixels were discarded. The results of the whole image segmentation procedure could be visually inspected by drawing the contours of the selected objects onto the original image.

**Figure 5:**
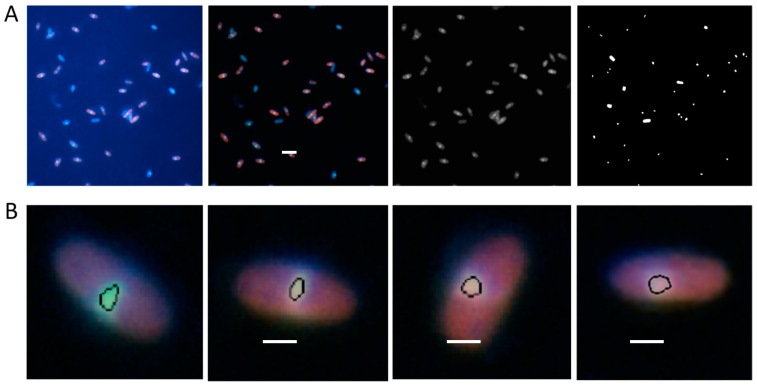
Image processing steps for nucleoid morphology analysis of *E. coli* C41(DE3) pLys expressing the Lpt peptide. (**A**) From left to right: raw fluorescence image captured with the UV2B filter; background subtracted image; grayscale image; segmented image objects representing the nucleoid. Scale bar 5 μm. (**B**) Zoom-in of selected cells showing the contours of the identified nucleoids. Scale bar 1 μm.

For nucleoid morphology measurements, the background-corrected color image was converted in grayscale and segmented by using the *imextendedmax* function with a H-maxima transform of 30 and a pixel connectivity of 8 (Figure 5A). As before, objects touching the edges or with an area larger than 2000 pixels or smaller than 50 pixels or with a perimeter higher than 250 pixels were discarded. To improve data accuracy, the contour of the selected objects was drawn onto the original color image for visual inspection. Mistaken nucleoid identifications were manually deleted with a mouse click. Nucleoid morphology parameters such as area, perimeter, circularity, and eccentricity could then be extracted from the selected objects (Figure 5B and [20]).

### 3.7. DAPI/EB Staining to Assess Membrane Integrity in Bulk Culture Samples

Although DAPI/EB staining provides information at the single cell level, often it is sufficient just to know if the toxin has been expressed, and if it has the expected phenotype, possibly in bulk culture samples. This is particularly important for bacterial toxins that are subjected to a rapid selection of inactive mutants. Taking advantage of the fact that both DAPI and EB can be efficiently excited by the UV light emitted from a standard laboratory UV-transilluminator, we verified the feasibility of this apparatus for detecting the expression and/or the degree of toxicity of a membrane-active peptide. *E. coli* C41 plysS cells freshly transformed with pET11b-*lpt* were grown on liquid media and 1 mL of culture was harvested before and after induction with IPTG. Cells were washed with PBS and stained with a DAPI/EB mix as described above. After removing the excess of dye, cells were resuspended in 50 μL of PBS and the tubes were exposed to the transilluminator UV light (λ max = 312 nm). As shown in Figure 4C, the red fluorescence of the induced sample confirmed expression and toxicity of the membrane-active peptide Lpt. This procedure could be very practical for a rapid check of toxin expression, however samples used for fluorescence microscopy should not be exposed to the transilluminator UV light because it may result in DAPI photobleaching.

### 3.8. Atomic Force Microscopy Imaging to Reveal Membrane Damages

Atomic force microscopy is an excellent tool for the investigation of several small hydrophobic peptides belonging to the antimicrobial peptide families. For instance, by recording high resolution topographic images, it is possible to analyze the mechanisms of action of antimicrobial peptides on supported lipid bilayers and on whole *E. coli* cells [37,38]. AFM imaging has also been used to monitor the formation of pores and membrane solubilization associated with caerin in living *Klebsiella pneumoniae* cells [39]. Recently, we employed AFM imaging to visualize the detrimental effect of the type I toxin Lpt on the outer membrane of *E. coli* cells after induction [20]. It should be noted that antimicrobial peptides are delivered in the culture medium, thereby their toxic effect is primarily exerted onto the outer membrane surface of gram-negative bacteria. Conversely, type I toxins are expressed within the cell, therefore their effect can be detected by the AFM probe only after translocation of the peptide from the inner to the outer membrane. To study the effect of type I toxins on the outer membrane, bacterial cells were prepared, starting from single colonies of freshly transformed *E. coli* C41 (DE3) pLysS cells grown overnight on solid media and cultured in LB liquid medium. At an OD_600 nm_ of 0.2, toxin expression was induced by the addition of 1 mM IPTG and cells were harvested after two hours from induction. Cells were washed three times with 1 mL of PBS and re-suspended in 50 μL of PBS. An aliquot of 25 μL of the PBS cell suspension was deposited onto a poly-L ornithine-functionalized coverslip for 30 s, followed by a gentle wash with Milli-Q water and dried by evaporation at room temperature. The glass coverslip was placed onto the AFM microscope and the region of interest was selected by means of the optical system. After engaging the AFM probe with the surface, the scan was started. The scan size was initially set to 20 × 20 μm to include 10–20 cell per view and successively reduced to 4 × 4 μm to resolve details of the cell surface. For better results, images were collected at a low scan rate (0.5 Hz) and with the maximum image size.

The high surface resolution of the AFM can also be combined with fluorescence imaging to gather complementary information on the cell status. In particular, with the DAPI/EB protocol described above, fluorescence imaging can provide a map of cells with impaired membrane permeability that can be used to guide AFM imaging. In this case, the sample preparation procedure must be adapted to include staining with fluorescence dyes. For instance, DAPI and EB, at a final concentration of 10 μg/mL each, can be added to the 50 μL cell suspension after the PBS wash, followed by 5-min incubation at room temperature, two washes with 1 mL of PBS to remove the excess of dye, and resuspension in 50 μL of PBS.

**Figure 6:**
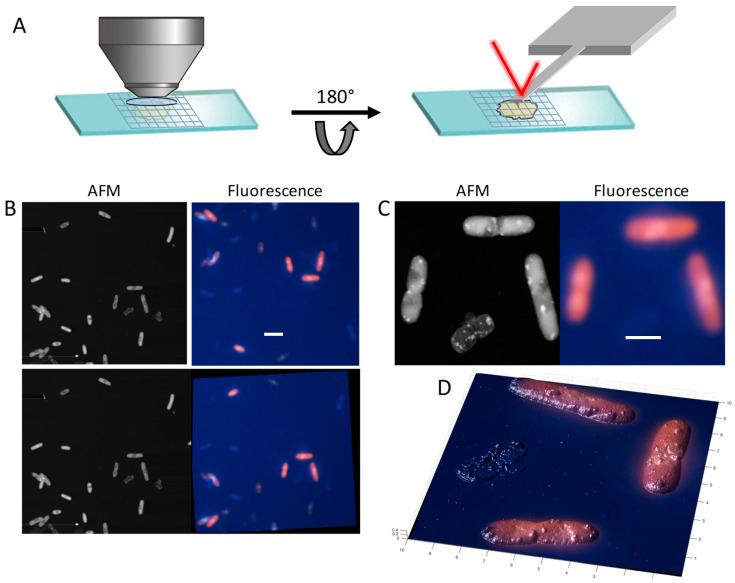
(**A**) Schematic representation of the combined AFM/fluorescence setup used to characterize *E. coli* C41 expressing the Lpt peptide. (**B**) Image registration carried out with the Matlab Image Registration app as described in the text. Scale bar 5 μm. (**C**) AFM image of *E. coli* cells expressing Lpt toxin obtained with a Park XE-100 microscope (**left**) and the registered fluorescence image (**right**). Scale bar 2 μm. (**D**) Three-dimensional topographic view of the AFM image with overlaid fluorescence signal. Membrane damaged cells maintain the rod-like shape of intact cells, however they appear deflated with protruding globular features scattered over the surface.

With a combined AFM/fluorescence microscope, imaging is straightforward, however, good results can also be obtained also with standalone AFM and fluorescence microscopes (Figure 6A). In this case, it is convenient to deposit the sample onto gridded glass coverslips to facilitate the finding of the field of observation. Because the sample must be moved from one microscope to the other, the sample is kept dried for better stability and consistency of results. The sample is first viewed under the fluorescence microscope where DAPI stained cell and EB stained cells are identified and mapped on the grid. Fluorescence images are stored for subsequent processing. The glass coverslip was recovered, the excess oil was removed, and the AFM analysis was carried out as described above, using the grid to locate the field of view analyzed with the fluorescence microscope. To obtain combined AFM/fluorescence images, we used the Matlab Image Registration app that provides an interactive UI environment to the *imregister* tool chain for image alignment (Figure 6B). First, the fluorescence image was cropped to the region represented by the AFM scan. Then the images were loaded into the app, and the parameters (method, transformation type, number of feature, quality of feature) were adjusted to obtain the best alignment between the two images. The geometric transformation (tform) provided by the app was applied to the cropped fluorescence color image by the *imwarp* function. This procedure can be repeated to improve alignment. The registered fluorescence and AFM images can be displayed side-by-side or overlaid to obtain a three-dimensional topographic view of the cells with the colored fluorescence signal (Figure 6C,D).

## 4. Conclusions

In vivo and in vitro studies of TA systems require cloning, expression, and purification of highly toxic proteins, thus special attention should be paid when choosing plasmid vectors, expression systems, and bacterial cell host. In our experience, the expression system pET can be used only in association with a vector carrying the T7 lysozyme, capable to down regulate transcription of T7 RNA polymerase, and with *E. coli* strains such as the C41 or C43 (DE3) pLysS that were specifically developed for the production of toxic polypeptides. At present, the proposed study protocols are limited to the use of the *E. coli* host, the pET system and to the use of antibiotics. Equivalent highly-repressed and inducible expression systems for Gram positive bacteria are not commercially available. The induced toxic phenotype of type I and type II toxins can be quantitatively evaluated by using fluorescence microscopy of cultured cells stained with dyes commonly used for detecting DNA and RNA. Atomic force microscopy may also provide useful information regarding the membrane damages caused by membrane-associated type I toxins. AFM can be performed either in air or under liquid. Generally, images of bacteria under liquid show a smooth surface, while bacteria imaged in air may show irregularities of the surface and a lower vertical dimension. However, imaging in air facilitates the use of a combined AFM/fluorescence setup because of the higher stability of the dried sample.
